# KIM-1 as an Early Diagnostic Biomarker of Cisplatin-Induced Acute Kidney Injury

**DOI:** 10.5812/ijpr-164432

**Published:** 2026-02-08

**Authors:** Ai-Hui Jin, Yan-Jie Zheng, Chen-Long Xu, Li Zhou, Hao Wang, Wen-Juan Wang

**Affiliations:** 1 Ningbo Hospital of Integrated Traditional Chinese and Western Medicine, Ningbo, China; 2 Ningbo College of Health Sciences, Ningbo, China

**Keywords:** Kidney Injury Molecule-1, Acute Kidney Injury, Cisplatin, Biomarkers, Proximal Tubules

## Abstract

**Background:**

Acute kidney injury (AKI) is a frequent complication of cisplatin chemotherapy, largely due to its toxic accumulation in renal proximal tubules. Current biomarkers show limited sensitivity in early detection, underscoring the need for more specific indicators. Kidney Injury Molecule-1 (KIM-1), upregulated in damaged tubular cells, shows promise as an early and accurate AKI biomarker.

**Objectives:**

This multimodal study integrates bioinformatic analyses, in vitro experiments, and a systematic review with meta-analysis to comprehensively evaluate KIM-1 expression, function, and early diagnostic performance.

**Methods:**

We analyzed single-cell RNA sequencing (scRNA-seq) and bulk RNA sequencing (RNA-seq) data to assess HAVCR1 expression and co-regulated genes in kidney tissue and cisplatin-treated HK-2 cells. Protein interactions were mapped using STRING and Cytoscape. In vitro assays, including quantitative polymerase chain reaction (qPCR), enzyme-linked immunosorbent assay (ELISA), and Cell Counting Kit-8 (CCK-8), were conducted on cisplatin-treated HK-2 cells. For the systematic review and meta-analysis, a Preferred Reporting Items for Systematic Reviews and Meta-Analyses (PRISMA)–guided search was conducted in PubMed, Scopus, Web of Science, Cochrane Library, and CNKI to identify studies evaluating urinary KIM-1 for early (≤ 24 h) detection of cisplatin-induced AKI. Study eligibility was based on clinical AKI defined by serum creatinine criteria, early KIM-1 measurement, and extractable 2 × 2 diagnostic data. Risk of bias was evaluated using the Quality Assessment of Diagnostic Accuracy Studies-2 (QUADAS-2) tool, and pooled estimates were generated using a bivariate model.

**Results:**

Single-cell and bulk RNA-seq analyses revealed that HAVCR1 (KIM-1) is specifically expressed in proximal tubular cells and is upregulated following cisplatin-induced injury. Gene enrichment and protein interaction analyses confirmed its association with epithelial transport and injury response pathways. In vitro, cisplatin treatment led to dose-dependent increases in KIM-1 mRNA and protein levels. Meta-analysis showed that urinary KIM-1 has acceptable diagnostic accuracy (AUC = 0.76; 95% CI: 0.65 - 0.86) as an early biomarker for cisplatin-induced AKI.

**Conclusions:**

KIM-1 is a proximal tubule–specific, injury-responsive biomarker that is upregulated early during cisplatin-induced kidney damage. Integrative bioinformatic and experimental analyses underscore its potential for early AKI detection, and the meta-analysis further supports its emerging clinical utility.

## 1. Background

Acute kidney injury (AKI) is characterized by a sudden elevation in serum creatinine, a reduction in urine output, or both, as defined by current clinical guidelines (1, 2). Acute kidney injury affects approximately 10 - 15% of hospitalized patients, with its prevalence rising to over 50% among those in intensive care units (1, 3-5). Strategies to predict and minimize AKI using patient-specific risk factors and machine learning models have recently been explored in other clinical contexts, such as cardiac surgery, providing insights that may be relevant for drug-induced nephrotoxicity (6). Cis-diamminedichloroplatinum (II), commonly known as cisplatin, is a platinum-based chemotherapeutic agent used in treating various cancers such as bladder, ovarian, lung, and testicular malignancies (7). It is administered to 10 - 20% of cancer patients due to its notable efficacy in slowing tumor progression. Cisplatin uptake and clearance are largely facilitated by transporters localized in renal proximal tubules, including OCT2 and MATE1. This leads to its accumulation in these cells, ultimately triggering inflammation, cellular injury, and death (8). Acute kidney injury represents the most significant dose-limiting side effect of cisplatin therapy, affecting up to 30% of treated individuals. Clinically, it is defined as a decline in kidney function, usually reflected by an increase in serum creatinine of ≥0.3 mg/dL or a decrease in urine output to ≤ 0.5 mL/kg/h (9). Biomarkers like neutrophil gelatinase-associated lipocalin (NGAL), kidney injury molecule-1 (KIM-1), and Cystatin-C are widely used for the detection of AKI in both clinical and preclinical contexts (10). However, these biomarkers may lack sensitivity in detecting mild renal injuries, and their expression can vary based on patient-specific factors including age (11), sex, and comorbid conditions (12, 13). Kidney injury molecule-1, also referred to as T-cell immunoglobulin and mucin domain-containing protein-1 (TIM-1) in humans and Kim-1/Tim-1 in rodents, is a type I transmembrane glycoprotein with a distinct extracellular Ig-like and mucin domain structure (14-16). It is recognized as a highly informative biomarker of renal injury (17). Functionally, KIM-1 acts as a phosphatidylserine receptor, promoting the recognition and phagocytosis of apoptotic cells by proximal tubular epithelial cells, which are thereby reprogrammed into semiprofessional phagocytes (15, 18). Kidney injury molecule-1 has demonstrated promise as an early urinary biomarker, capable of detecting AKI within 24 hours post-injury, with particular utility in identifying ischemic acute tubular necrosis (19, 20). Despite its promise, the early diagnostic role of KIM-1 specifically in cisplatin-induced AKI remains insufficiently characterized, and the molecular context underlying its induction is not fully defined.

## 2. Objectives

This multi-modal study integrates transcriptomic profiling, in vitro experiments, and a clinical diagnostic systematic review and meta-analysis to comprehensively evaluate KIM-1 as an early biomarker of cisplatin-associated AKI.

## 3. Methods

### 3.1. Single-Cell RNA-Sequencing and Cellular Localization Analysis

To investigate the cell-type specificity of HAVCR1 (the gene encoding KIM-1) and identify co-expressed genes, we analyzed normal human kidney single-cell RNA sequencing (scRNA-seq) data from the Human Protein Atlas (HPA) (https://www.proteinatlas.org/). Cell clusters were annotated using known marker genes, and the top 15 transcriptional neighbors of HAVCR1 were identified based on expression pattern similarity across proximal tubular cells. Cellular localization of HAVCR1 was determined using protein annotation from UniProt (https://www.uniprot.org). To assess HAVCR1 dynamics in acute injury, we used the Susztak Lab Kidney Biobank (http://www.susztaklab.com/) interactive scRNA-seq dataset from a mouse model of ischemia-reperfusion injury (21).

### 3.2. Bulk RNA-Sequencing Analysis and Gene Set Enrichment

RNA sequencing (RNA-seq) data from the GSE227970 dataset, containing HK-2 cells exposed to cisplatin and untreated controls for 48 hours, were analyzed through GEO2R to identify differentially expressed genes (DEGs). Genes showing a log₂ fold change of ≥1 and an adjusted P-value below 0.05 were considered significant. To illustrate overall gene expression alterations, a volcano plot was created. Gene Set Enrichment Analysis (GSEA) was performed using version 4.4.0 of the GSEA tool, and the resulting enrichment data were visualized using the GseaVis package in R (22).

### 3.3. Protein–Protein Interaction Network and Hub Analysis

A protein–protein interaction network was constructed using genes from the HAVCR1 Neighborhood Signature via the STRING database and visualized in Cytoscape (v3.9.1). Interactions were filtered using a medium confidence score (≥ 0.4). The CytoHubba plugin was used to assess network topology, and the bottleneck algorithm was applied to identify central hub genes. Node connectivity and ranking were visualized using a color gradient.

### 3.4. Cell Culture and Cisplatin Treatment

HK-2 cells, a human proximal tubular epithelial cell line (Zhongqiaoxinzhou Biotechnology Co., Ltd., Shanghai, China), were cultured in DMEM/F12 medium supplemented with 10% fetal bovine serum (FBS), 100 U/mL penicillin, and 100 μg/mL streptomycin. Cells were maintained at 37°C in a humidified atmosphere containing 5% CO₂. For treatment experiments, after overnight incubation to allow cell attachment, cisplatin (MCE, HY-17394) was applied at the designated concentrations for subsequent assays.

### 3.5. Cell Viability Assay

Cell viability was assessed using the Cell Counting Kit-8 (CCK-8) assay (Beyotime, China) according to the manufacturer’s instructions. HK-2 cells were plated in 96-well plates, allowed to adhere overnight, and then treated with cisplatin at concentrations of 5, 10, and 20 μM. Cell viability was measured at 0, 12-, 24-, 48-, and 72-hours post-treatment. At each time point, 10 μL of CCK-8 reagent was added to each well and incubated at 37°C for 2 hours, after which absorbance at 450 nm was recorded using a microplate reader. Absorbance values were normalized to the untreated control group, and cell viability was calculated as: Viability (%) = (ODtreated / ODcontrol) × 100.

### 3.6. Quantitative Real-Time Polymerase Chain Reaction Analysis in Cell Culture

Total RNA was isolated from HK-2 cells using the RNAsimple Total RNA Kit (TIANGEN), in accordance with the manufacturer’s instructions. RNA concentration and purity were determined using a BioDrop spectrophotometer. For complementary DNA (cDNA) synthesis, 500 ng of RNA was reverse transcribed with the HiFiScript cDNA Synthesis Kit (Cowin Century Biotechnology). Quantitative real-time polymerase chain reaction (qRT-PCR) was conducted using the NovoStart^®^ SYBR qPCR SuperMix Plus kit (Shanghai Nearshore Technology) on an Applied Biosystems QuantStudio 7 Flex system. Each 10 µL polymerase chain reaction included 2× SuperMix, gene-specific primers, cDNA, and RNase-free water. The thermal cycling conditions consisted of an initial denaturation at 95°C for 30 seconds, followed by 40 cycles of 95°C for 5 seconds and 60°C for 30 seconds. All reactions were performed in triplicate, with β-Actin used as the internal control. Relative gene expression was calculated using the 2^-ΔΔCt^) method, using untreated cells as the reference group. Primer sequences were as follows:

HAVCR1: F: 5′-TGGCAGATTCTGTAGCTGGTT-3′, R: 5′-AGAGAACATGAGCCTCTATTCCA-3′; SLC17A1: F: 5′-ACCCTATGTATAATTGGAGCCCA-3′, R: 5′-GGATGAGCAGGCTTAACACAGA-3′; SLC17A3: F: 5′-CTTTACTGCCATCCTCATAGGTG-3′, R: 5′-AGACCCGACCTGTTGTTTCAA-3′; β-Actin: F: 5′-ATAGCACAGCCTGGATAGCAACGTAC-3′, R: 5′-CACCTTCTACAATGAGCTGCGTGTG-3′.

### 3.7. Enzyme-Linked Immunosorbent Assay

The concentration of secreted KIM-1 in HK-2 cell culture supernatants was measured using a human KIM-1 enzyme-linked immunosorbent assay (ELISA) kit (FineTest, EH0210, Wuhan, China) following the manufacturer’s instructions. Supernatants were collected from cells treated with various concentrations of cisplatin (including untreated controls), centrifuged, and either analyzed immediately or stored at -80°C. Samples and standards were added to antibody-coated wells, followed by incubation with biotin-labeled detection antibody, horseradish peroxidase (HRP)-streptavidin, and 3,3′,5,5′-tetramethylbenzidine (TMB) substrate. Absorbance was read at 450 nm, and KIM-1 levels were calculated from a standard curve and reported as fold change relative to the untreated control.

### 3.8. Systematic Review and Meta-Analysis

Following the Preferred Reporting Items for Systematic Reviews and Meta-Analyses (PRISMA) guidelines (23), a comprehensive literature search was performed across Web of Science, Scopus, the Cochrane Library, MEDLINE/PubMed, and the CNKI up to May 20, 2025, without language restrictions. In MEDLINE/PubMed, a combination of Medical Subject Headings (MeSH) terms, Supplementary Concepts, and free-text keywords was used, including: ("HAVCR1"[Text Word] OR "Kidney Injury Molecule-1"[Text Word] OR "KIM-1"[Text Word]) AND ("Acute Kidney Injury"[MeSH Terms] OR "acute kidney injury"[Text Word] OR AKI[Text Word]) AND ("Cisplatin"[MeSH Terms] OR cisplatin[Text Word]). For the other databases, equivalent free-text terms were applied, such as: ("KIM-1" OR "Kidney Injury Molecule-1") AND ("acute kidney injury" OR "acute renal injury") AND ("cisplatin" OR "cisplatin nephrotoxicity"). The full electronic search strategies for all databases are provided in Appendix 1 in Supplementary File. To ensure completeness, reference lists of included articles, relevant review papers, and related records identified through Google Scholar were also screened. Duplicate records were removed using EndNote software (Version 21, Thomson Reuters). Two independent reviewers independently screened the titles and abstracts of all retrieved records to assess their eligibility. Studies were included based on the following criteria: (1) Enrolled human cases at risk of AKI specifically due to cisplatin exposure; (2) used serum creatinine as the reference standard for AKI diagnosis, with AKI defined according to established clinical criteria from either the Acute Kidney Injury Network (AKIN) or the Kidney Disease: Improving Global Outcomes (KDIGO) guidelines; (3) evaluated KIM-1 as a urine biomarker specifically for the early detection of AKI, defined as measurement within 24 hours after cisplatin administration; and (4) provided adequate data to build a 2 × 2 contingency table (true negatives, false negatives, true positives, and false positives). Studies were excluded if they: (1) Assessed AKI caused by nephrotoxic agents other than cisplatin; (2) did not evaluate KIM-1 for early AKI detection; (3) were review articles, editorials, conference abstracts, case reports, animal studies, in silico studies, in vitro studies, or duplicate publications; or (4) lacked sufficient diagnostic data for 2 × 2 table construction. Data from each included study were extracted using a structured data extraction form. For every eligible publication, we collected the following key study characteristics: First author, year of publication, country of origin, study design, total number of AKI and non-AKI participants, mean age, percentage of male participants, and the analytical method used to quantify urinary KIM-1 (e.g., ELISA). Additional clinically relevant variables, including the reported KIM-1 cut-off value (ng/mg creatinine), baseline estimated glomerular filtration rate (eGFR), and cumulative cisplatin dose, were also extracted. The methodological quality of the involved studies was appraised using the quality assessment of diagnostic accuracy studies-2 (QUADAS-2) tool, which focuses on four critical domains: Patient selection, index test, reference standard, and flow and timing. Each domain was assessed for potential risk of bias, and the first three domains were also evaluated for applicability concerns. Ratings for each item were categorized as low, high, or unclear risk, and any discrepancies between reviewers were resolved through discussion until consensus was reached. To enable quantitative synthesis of diagnostic accuracy, we extracted or reconstructed the 2 × 2 contingency matrix for each study, including the number of true positives, false positives, true negatives, and false negatives for early AKI detection. A standard bivariate meta-analytic model was applied to compute pooled diagnostic metrics, including sensitivity, specificity, pooled positive likelihood ratio (PPLR), pooled negative likelihood ratio (PNLR), and diagnostic odds ratio (DOR). To evaluate the overall diagnostic accuracy of KIM-1, hierarchical summary receiver operating characteristic (HSROC) curves were plotted, and the area under the curve (AUC) was calculated. Area under the curve values were interpreted as follows: 0.50 - 0.70, 0.71 - 0.79, 0.80 - 0.89, and 0.90 - 1.00 show unacceptable, acceptable, good, and excellent, respectively (24, 25). In the meta-analysis section of the present study, statistical calculations were performed and summarized with 95% confidence intervals (95% CI). The meta-analysis was carried out using Meta-DiSc version 2 (available at: https://ciberisciii.shinyapps.io/MetaDiSc2/) (26) and RevMan version 5.4 software.

### 3.9. Statistical Analysis

All statistical analyses for in vitro experiments were performed using GraphPad Prism (version 8). Data are presented as mean ± standard deviation (SD). For the viability assay, differences among treatment groups across time points were analyzed using two-way analysis of variance (ANOVA) followed by Tukey’s post-hoc multiple comparisons test. For quantitative polymerase chain reaction and enzyme-linked immunosorbent assay experiments, comparisons across cisplatin concentrations were performed using one-way ANOVA with Tukey’s post-hoc test. All experiments were conducted in three independent biological replicates (n = 3 per group). Statistical significance was defined as P < 0.05.

## 4. Results

### 4.1. HAVCR1 Is a Proximal Tubule-Localized, Cell Surface Marker Associated with Epithelial Transport Functions

**Figure 1.**
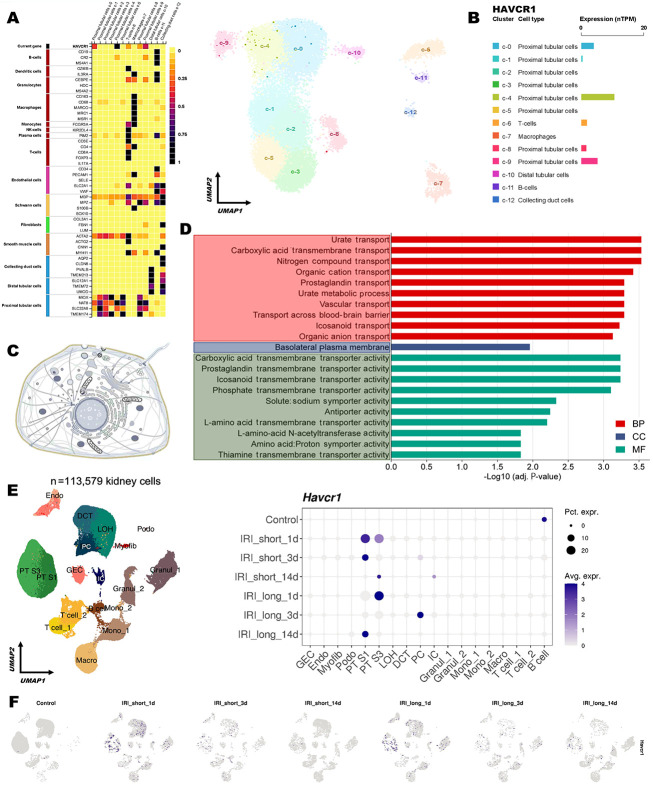
Proximal tubule-specific expression and early induction of HAVCR1 (KIM-1) in kidney injury models; A, left: Heatmap showing the expression of canonical marker genes used to define major kidney cell types. Right: UMAP projection of single-cell RNA-sequencing data from the Human Protein Atlas displaying 12 annotated kidney cell clusters; B, cluster-level bar plot showing HAVCR1 expression across kidney cell clusters, quantified as normalized transcript levels (nTPM). HAVCR1 expression is predominantly enriched in proximal tubular epithelial cell clusters (C0, C4, and C9); C, cellular localization of HAVCR1, identified as a type I transmembrane protein on the cell surface based on UniProt annotation; D, gene Ontology enrichment analysis of the HAVCR1 Neighborhood Signature (15 genes co-expressed with HAVCR1 in proximal tubular cells), performed using over-representation analysis. The bar plot displays the -log10 adjusted P-values for gene ontology terms that reached statistical significance (adjusted P < 0.05). Enriched categories span the three major Gene Ontology domains: Biological process, cellular component, and molecular function; E, UMAP visualization (left) of 113,579 mouse kidney cells, showing major renal cell populations. Samples included: Control (n = 6), IRI_short_1d (n = 2), IRI_short_3d (n = 2), IRI_short_14d (n = 2), IRI_long_1d (n = 2), IRI_long_3d (n = 2), and IRI_long_14d (n = 2). The dot plot (right) shows Havcr1 expression, where dot size indicates the percentage of expressing cells and color intensity reflects the average expression level. Havcr1 induction is predominantly observed in proximal tubule cells, with peak expression at day 1 following ischemia–reperfusion injury; F, UMAP feature plots show Havcr1 expression under control and ischemia–reperfusion injury conditions over time. Expression peaks at the first day post-injury and declines by days 3 and 14.

To investigate the expression pattern and potential role of HAVCR1 (KIM-1) in the kidney, we first examined its cell-type specificity using single-cell RNA sequencing (scRNA-seq) data from the Human Protein Atlas (HPA) (Figure 1A). A total of 12 distinct cell clusters were identified, representing major nephron segments and immune cells. These clusters were annotated using canonical marker genes (Figure 1A, left), and their transcriptional relationships were visualized using a UMAP projection (Figure 1A, right). HAVCR1 expression was predominantly localized to proximal tubular epithelial cells, specifically within clusters C0, C4, and C9, as shown in the cluster-level bar plot (Figure 1B). To determine its cellular localization, we obtained annotation data from the UniProt database, which lists HAVCR1 as a type I transmembrane glycoprotein located on the cell surface (Figure 1C). This supports its potential utility as an accessible biomarker and therapeutic target in renal injury. To further explore the transcriptional context of HAVCR1 in proximal tubular cells, we identified its 15 nearest neighbors based on expression pattern similarity in proximal tubular cell types, as reported by the HPA scRNA-seq browser. These genes include FCAMR, SLC22A2, SLC47A2, CLTRN, ENSG00000275163, SLC17A3, SLC22A8, WDR72, CLEC18C, NAT8, SLC17A1, CCDC198, SLC13A3, CDH6, and SLC36A2, forming a 16-gene set referred to as the HAVCR1 Neighborhood Signature. Gene Ontology enrichment analysis of this set revealed strong associations with biological processes such as urate transport, carboxylic acid transmembrane transport, organic cation and anion transmembrane transport, prostaglandin transport, and nitrogen compound transport. Enriched cellular components included the basolateral plasma membrane, while dominant molecular functions involved carboxylic acid transmembrane transport activity, solute:sodium symporters, antiporters, and different amino acid transporters (Figure 1D). These results suggest that HAVCR1 is part of a broader renal epithelial transport program.

### 4.2. Early HAVCR1 Expression Reveals Proximal Tubule-Specific Response to Cisplatin-Induced Injury

**Figure 2.**
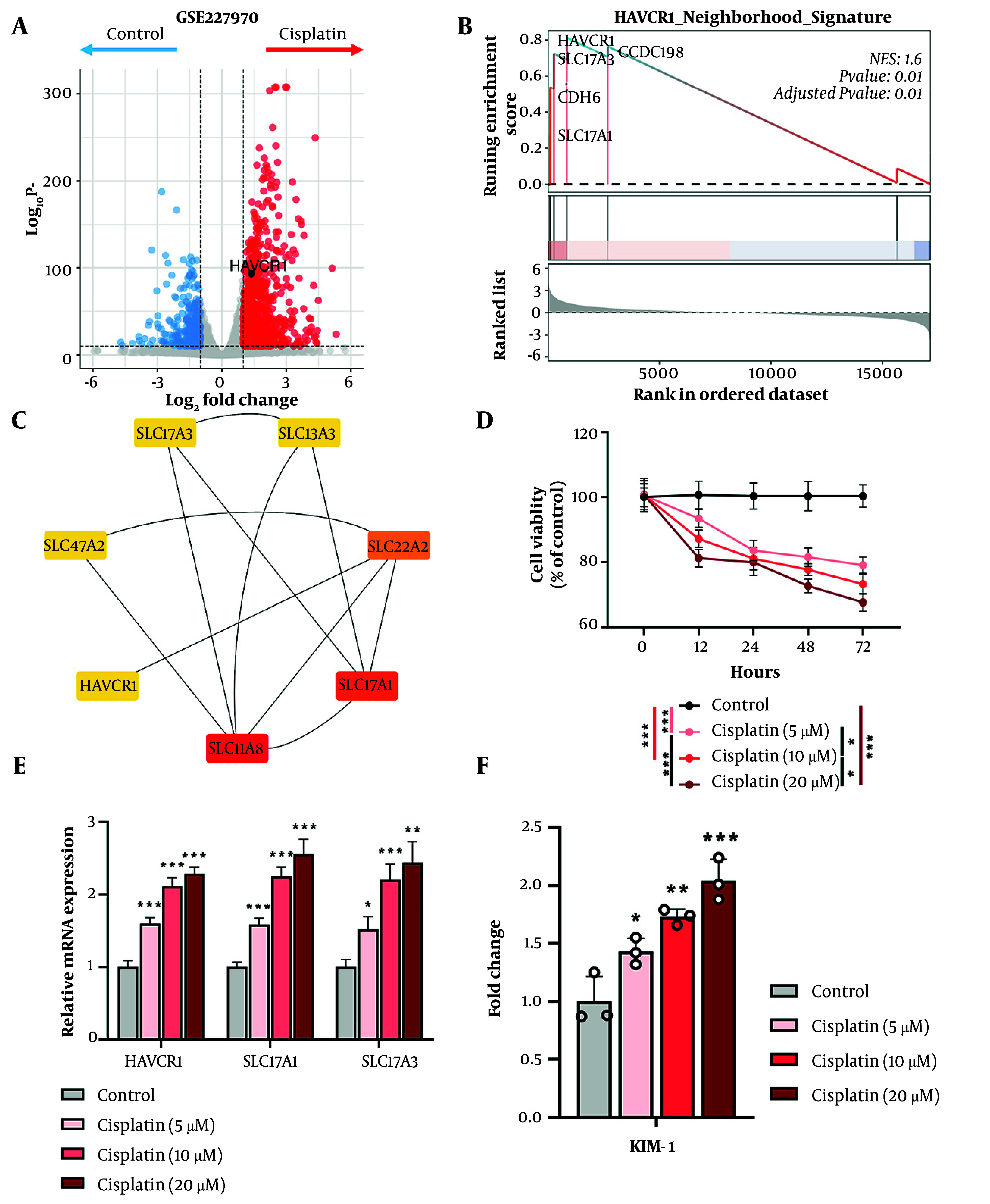
HAVCR1/Kidney Injury Molecule-1 (KIM-1) is transcriptionally and functionally induced by cisplatin exposure; A, volcano plot from RNA sequencing data (GSE227970) shows significant upregulation of HAVCR1 in cisplatin-treated HK-2 cells; B, gene Set Enrichment Analysis reveals enrichment of the HAVCR1 Neighborhood Signature (NES = 1.6, adjusted P = 0.01), indicating coordinated activation of proximal tubule injury-response genes; C, protein–protein interaction network generated from the HAVCR1 Neighborhood Signature using STRING and visualized in Cytoscape. Of the 15 genes in the signature, seven displayed interaction evidence in STRING at the predefined medium confidence threshold (≥ 0.4) and were therefore included in the network. Node color represents bottleneck centrality scores, with SLC22A8 identified as the top-ranked hub gene; D, cisplatin decreases HK-2 cell viability in a dose-dependent manner over 72 hours. Cell viability was calculated as the percentage of absorbance relative to untreated control cells at each time point. Data represent three independent biological replicates (n = 3 per group) and are shown as mean ± SD. Statistical analysis was performed using two-way analysis of variance followed by Tukey’s post-hoc multiple comparisons test. Significance is indicated as *P < 0.05, and ***P < 0.001; E, Bar plot of quantitative polymerase chain reaction results indicates a dose-dependent increase in HAVCR1, SLC17A1, and SLC17A3 mRNA expression following cisplatin treatment. Data represent three independent experiments (n = 3 per group) and are presented as mean ± SD. Statistical analysis was performed using one-way analysis of variance followed by Tukey’s post-hoc multiple comparisons test. Significance is indicated as *P < 0.05, **P < 0.01, and ***P < 0.001; F, enzyme-linked immunosorbent assay measurement of secreted KIM-1 protein shows a dose-dependent increase following cisplatin treatment. Data represent three independent experiments (n = 3 per group) and are shown as mean ± SD. Statistical analysis was performed using one-way analysis of variance followed by Tukey’s post-hoc multiple comparisons test. Significance is indicated as *P < 0.05, **P < 0.01, and ***P < 0.001.

To further assess HAVCR1 expression dynamics during AKI, we analyzed a publicly available single-cell RNA sequencing (scRNA-seq) dataset from a mouse model of ischemia-reperfusion injury (IRI), a widely used in vivo model for studying AKI. The dataset, retrieved from the Susztak Lab Kidney Biobank [http://www.susztaklab.com/; Balzer et al., Nat Commun, (21)], contains 113,579 single cells from mouse kidneys subjected to short (23 min) or long (30 min) bilateral IRI, collected at 1-, 3-, and 14-days post-injury, along with sham-operated controls. Consistent with prior findings, HAVCR1 expression was minimal in control kidneys. Following both short and long IRI, HAVCR1 expression increased markedly, particularly in proximal tubular cells, indicating a strong injury-induced response. Notably, the most dramatic increase occurred at day 1 post-injury, with expression levels declining by days 3 and 14, suggesting HAVCR1 may serve as a sensitive early biomarker of kidney injury (Figure 1E). UMAP-based visualization further confirmed its localization to proximal tubule clusters post-injury (Figure 1F). To explore whether a similar transcriptional response occurs under nephrotoxic stress in vitro, we analyzed bulk RNA sequencing (RNA-seq) data from GSE227970, which profiled HK-2 cells — a human proximal tubule cell line — treated with cisplatin. Differential expression analysis revealed substantial transcriptional changes following cisplatin treatment, with numerous genes significantly dysregulated. Importantly, HAVCR1 was strongly upregulated in the cisplatin-treated group, consistent with its known association with proximal tubule injury (Figure 2A). To evaluate whether HAVCR1 functions as part of a coordinated gene program in this injury context, we performed Gene Set Enrichment Analysis (GSEA) on the GSE227970 dataset using the previously defined HAVCR1 Neighborhood Signature. The analysis revealed significant enrichment in the cisplatin-treated group (NES = 1.6, adjusted P-value = 0.01), indicating transcriptional activation of this module under acute toxic injury conditions (Figure 2B). Among the genes contributing most to the enrichment were HAVCR1, SLC17A1, SLC17A3, CDH6, and CCDC198, supporting the view that this gene set reflects a proximal tubular epithelial response to cisplatin-induced stress. To further contextualize these findings and examine the functional relationships among HAVCR1 and its associated genes, we next constructed a protein–protein interaction network using the Cytoscape platform with data sourced from the STRING database. This analysis focused on the genes from the HAVCR1 Neighborhood Signature that were connected through experimentally supported or predicted interactions. As shown in Figure 2C, seven genes formed a coherent network, which we evaluated using the CytoHubba plugin and the bottleneck centrality algorithm to identify key hub genes. The remaining genes lacked validated or predicted protein–protein interactions and were therefore not included in the protein–protein interaction network visualization. Within this network, SLC22A8, SLC17A1, and SLC22A2 ranked as the most central nodes, with HAVCR1 occupying a core position in the connected module. Notably, SLC17A1 and SLC17A3, along with HAVCR1, were also among the top-contributing genes in the Gene Set Enrichment Analysis enrichment analysis (Figure 2B), suggesting that these genes not only respond robustly to cisplatin exposure at the transcriptomic level but are also functionally integrated within the same injury-associated proximal tubule network. The overlap across both network topology and enrichment analysis underscores their potential cooperative role in the transcriptional response to cisplatin-induced epithelial stress.

### 4.3. in vitro Validation Reveals Early Dose-Dependent Induction of HAVCR1 by Cisplatin in Human Proximal Tubular Cells

To functionally validate the injury-associated transcriptional activation of HAVCR1 and its related genes, we performed in vitro cytotoxicity assays in HK-2 cells. Cells were treated with increasing concentrations of cisplatin (5, 10, and 20 μM), and cell viability was quantified at multiple time points (0, 12, 24, 48, and 72 hours). As shown in Figure 2D, cisplatin induced a clear, dose-dependent decline in cell viability when expressed as a percentage of untreated control cells, confirming that the chosen drug concentrations elicited early and measurable cytotoxic injury in proximal tubular cells. This verification provided the necessary functional context for interpreting subsequent HAVCR1 mRNA and protein upregulation under identical treatment conditions. To validate gene expression changes, we next performed quantitative polymerase chain reaction analysis for HAVCR1, as well as two of its key network-associated transporters, SLC17A1 and SLC17A3, which were identified as central nodes in the upstream protein–protein interaction network analysis and among the top contributors in the Gene Set Enrichment Analysis enrichment results. All three genes exhibited a significant, dose-dependent increase in expression following cisplatin exposure, with the strongest induction observed at 20 μM (Figure 2E). These results validate their transcriptional responsiveness under nephrotoxic conditions. In parallel, we measured the protein secretion of KIM-1 (encoded by HAVCR1) in culture supernatants using enzyme-linked immunosorbent assay. Consistent with gene expression patterns, a significant, dose-dependent increase in secreted KIM-1 was observed (Figure 2F), confirming that HAVCR1 is not only transcriptionally activated but also translated and secreted in response to cisplatin injury. This supports its role as a functional biomarker of proximal tubule damage. Together, these findings demonstrate that HAVCR1 (KIM-1) is a proximal tubule-specific, injury-responsive gene that is rapidly induced following early exposure to cisplatin in both in vivo and in vitro models of AKI. Its prompt transcriptional and translational upregulation, along with co-expression with solute transporters and dose-dependent secretion into the extracellular environment, underscores its potential role as a sensitive and functional biomarker of early tubular stress. Based on these data, we next sought to evaluate the broader utility of KIM-1 as an early diagnostic indicator of cisplatin-induced nephrotoxicity through systematic meta-analysis.

### 4.4. Meta-Analysis Results Indicated That Urine KIM-1 Is an Early Biomarker of Cisplatin-Induced Acute Kidney Injury

**Figure 3.**
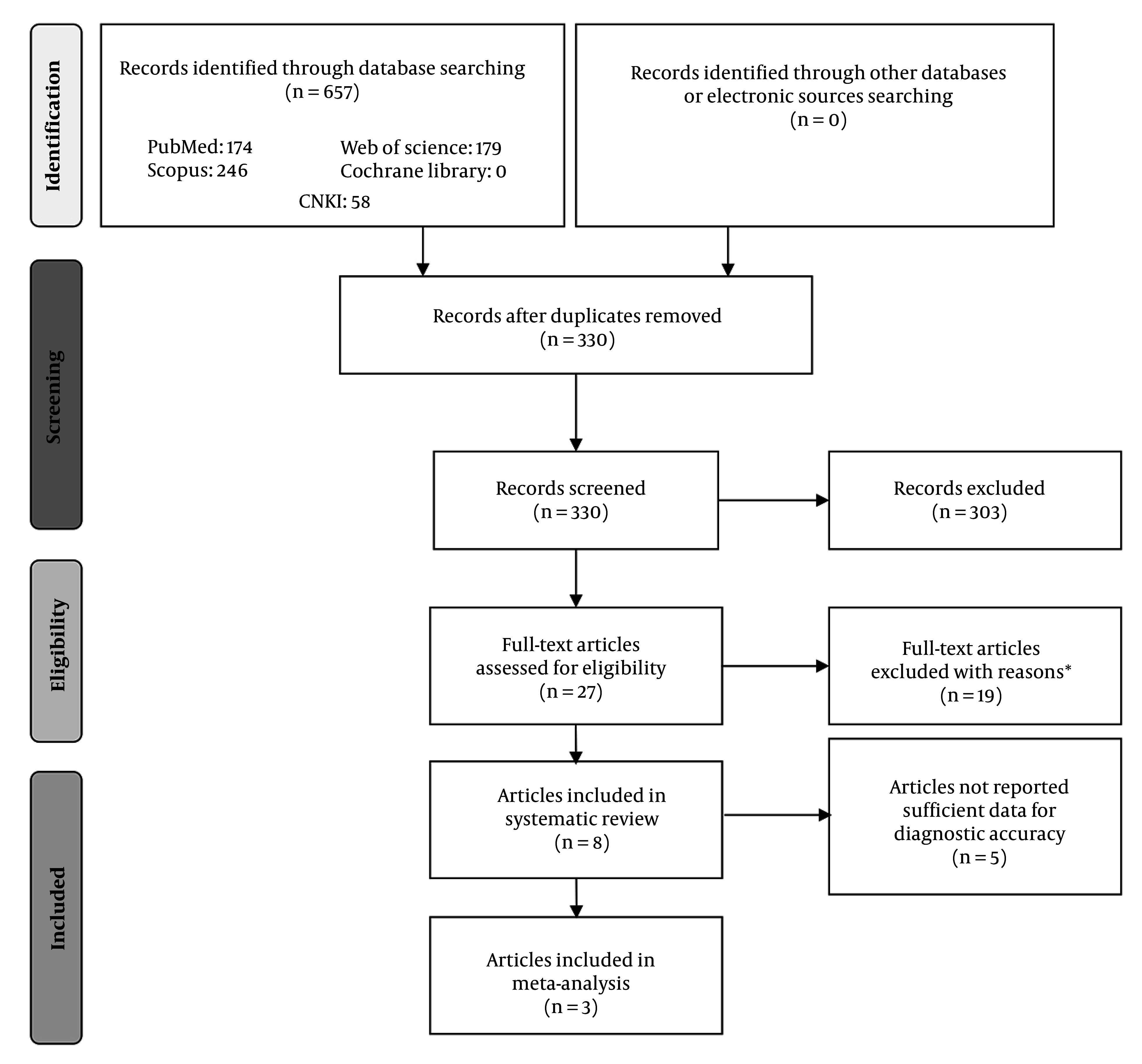
PRISMA flowchart of study selection process

Out of 657 initial records, 327 duplicate studies were removed, and 303 were excluded after screening titles and abstracts. The primary reasons for exclusion at this stage included (1) editorials, case reports, and clinical guidelines; (2) studies investigating AKI caused by nephrotoxic agents other than cisplatin; and (3) animal studies. Subsequently, 27 articles were assessed in full text. Of these, 19 were excluded for reasons such as (1) theses or dissertations, (2) review articles, and (3) lack of assessment of KIM-1 following early cisplatin exposure (within 24 hours). Ultimately, eight studies were included in the systematic review, but five lacked sufficient data to build 2 × 2 contingency tables, resulting in three studies being eligible for meta-analysis (Figure 3). Table 1 reports characteristics of the studies entered into the systematic review and then Table 2 summarizes the main features of the included studies in the meta-analysis. 

**Table 1.** Studies Reporting Urinary Kidney Injury Molecule-1 Level in Cisplatin-Induced Acute Kidney Injury Patients

First Author, Publication Year	Country	Design	Population Settings Treated with Cisplatin- Based Chemotherapy	AKI Definition	Sample Size	AKI Patients	Age (y)	Males, N (%)	Baseline eGRF (mL/ min/1.73 m^2^)	Baseline Serum Creatinine, μmol/L	Cisplatin Dose, mg/m^2^
**Zhu et al., 2023 (** 27 **)**	China	Retrospective cohort	Patients with various types of malignancies	Increase in creatinine from baseline to peak of ≥ 0.3 mg/dL	282	97	57.89	145 (51.4)	110.30	63	90
**Miloševski-Lomić et al., 2025 (** 28 **)**	Serbia	Cross-sectional study	Patients with various types of malignancies	KDIGO criteria	13	2	5	25 (69.4)	123.69	44.77	80 - 100 and then 20
**Shinke et al., 2015 (** 29 **)**	Japan	Cross-sectional study	Patients with lung cancer	KDIGO criteria	11	NR	65.9	7 (63.6)	NR	66.32	80, 60, or 64
**Szumilas et al., 2024 (** 30 **)**	Poland	Case-control study	Patients with various types of malignancies	KDIGO criteria	21	4	56	14 (66.7)	NR	57.47	69
**Tekce et al., 2015 (** 31 **)**	Turkey	Prospective cohort	Patients with gastric or lung tumors	AKIN criteria	22	8	57.32	16 (72.73)	102.91	81.72	75
**Pavkovic et al., 2016 (** 32 **)**	USA	Cohort	Patients with malignant mesothelioma undergoing cytoreductive surgery	AKIN criteria	106	45	63.88	81 (76.41)	NR	NR	NR
**Ghadrdan et al., 2020 (** 33 **)**	Iran	Cohort	Patients with various types of malignancies	AKIN criteria	35	7	51.83	25 (71.43)	110.48	80.29	181.14
**McMahon et al., 2022 (** 34 **)**	Canada	Prospective cohort	Patients with various types of malignancies	KDIGO criteria	148	43	6.82	143 (50)	145.19	NR	99.63

Abbreviations: AKI, acute kidney injury; KIM-1, kidney injury molecule-1.

**Table 2.** Features of Studies Entered Into the Meta-Analysis

Study ID	Country	Design	AKI/nAKI	Age	% Male	Method	Cut-off (ng/mg)	eGFR (mL/min/1.73 m^2^)	Culminative Cisplatin Dosage (mg)
**Ghadrdan et al., 2020 (** 33 **)**	Iran	Cohort	7/28	51.83	71.43	ELISA	1.38	110.49	181.14
**McMahon et al., 2022 (** 34 **)**	Canada	Cohort	43/105	6.82	50	ELISA	0.573	145.19	99.63
**Tekce et al., 2015 (** 31 **)**	Turkey	Cohort	8/14	57.32	72.73	ELISA	0.510	102.91	75

Abbreviations: AKI, acute kidney injury; nAKI, non-AKI; eGFR, estimated glomerular filtration rate.

**Figure 4.**
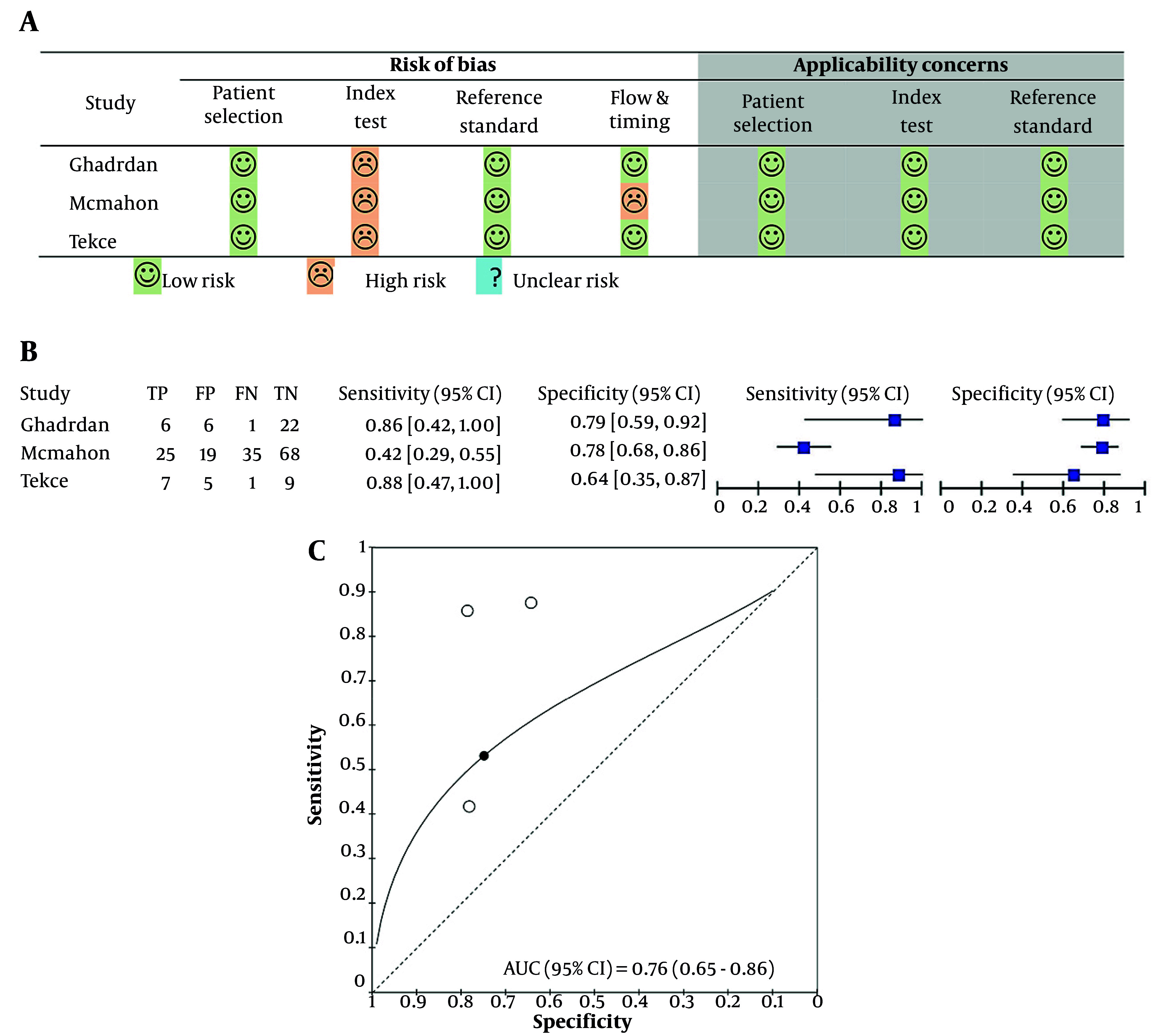
Meta-analysis evaluating the diagnostic performance of urine kidney injury molecule-1 (KIM-1) for early detection of cisplatin-induced AKI; A, quality evaluation of involved studies using the QUADAS-2 tool, illustrating risk of bias and applicability concerns; B, forest plots showing sensitivity and specificity of urine KIM-1 normalized by urine creatinine in each included study; C, summary receiver operating characteristic (ROC) curve and area under the curve (AUC) illustrating overall diagnostic accuracy.

The quality of the included studies was evaluated by the Quality Assessment of Diagnostic Accuracy Studies-2 (QUADAS-2) tool, with the results visually summarized for each study (Figure 4A). 

The assessment identified a high risk of bias in the "index test" domain, mainly because of the lack of a prespecified threshold. Nevertheless, no applicability concerns were noted for the studies evaluating KIM-1. Meta-analysis showed that the pooled sensitivity of KIM-1, standardized by urinary creatinine, was 0.53 (95% CI: 0.36 - 0.68), and the pooled specificity was 0.748 (95% CI: 0.625 - 0.841) (Table 3 and Figure 4B). The pooled diagnostic odds ratio (DOR) was 3.36 (95% CI: 1.69 - 6.68), indicating a modest ability to discriminate between AKI and non-AKI cases (Table 3). The hierarchical summary receiver operating characteristic (HSROC) analysis demonstrated an area under the curve (AUC) of 0.76 (95% CI: 0.65 - 0.86), which falls within the “acceptable” range for diagnostic performance and supports the potential of KIM-1 as an early biomarker for cisplatin-induced AKI (Figure 4C). However, due to the limited number of included studies (n = 3), subgroup analyses and meta-regression could not be performed.

**Table 3.** Diagnostic Accuracy of Urine Kidney Injury Molecule-1 for Early Detection of Acute Kidney Injury

Diagnostic Accuracy Metric	Pooled Estimate (95% CI)
**Pooled sensitivity **	0.53 (0.36 - 0.68)
**Pooled specificity **	0.74 (0.62 - 0.84)
**Pooled positive-likelihood ratio **	2.10 (1.38 - 3.21)
**Pooled negative-likelihood ratio **	0.62 (0.45 - 0.87)
**Diagnostic odds ratio **	3.36 (1.69 - 6.68)
**AUC **	0.76 (0.65 - 0.86)

## 5. Discussion

Acute kidney injury caused by cisplatin remains difficult to detect in its earliest stages because traditional biomarkers such as serum creatinine increase only after substantial tubular damage has occurred (19, 20). Although KIM-1 has been proposed as an early tubular injury marker, its early dynamics following cisplatin exposure have not been comprehensively evaluated across molecular, experimental, and clinical layers. Previous studies typically examined KIM-1 in isolated contexts, leaving uncertainty regarding its mechanistic role, cell-type specificity, and true diagnostic performance in humans (20). To address this gap, our study used an integrated, multi-level approach — combining single-cell transcriptomics, bulk RNA sequencing, in vitro functional assays, and a clinical systematic review with meta-analysis — to generate a unified and rigorous evaluation of HAVCR1/KIM-1 as an early indicator of cisplatin-induced kidney injury. Across these complementary analyses, we showed that KIM-1 is a proximal tubule–specific, injury-responsive molecule that becomes rapidly and strongly upregulated following nephrotoxic stress. Single-cell profiling established its precise cellular localization and transcriptional neighborhood, bulk RNA sequencing demonstrated its induction by cisplatin, and in vitro assays confirmed an early and dose-dependent induction of KIM-1 mRNA and protein expression, accompanied by a measurable reduction in cell viability within the first 24 – 48 hours of exposure. Importantly, the diagnostic meta-analysis demonstrated that urinary KIM-1 — as normalized to creatinine — provides a balanced sensitivity and specificity with acceptable diagnostic accuracy, reflected by an AUC of 0.76, supporting its potential value as an early, clinically applicable biomarker for cisplatin-associated acute kidney injury.

Cisplatin-induced acute kidney injury has been reported in approximately one-third of patients receiving this chemotherapeutic agent (35). The underlying nephrotoxic effects are attributed to multiple molecular mechanisms, including increased cellular uptake and accumulation in renal tissues, activation of inflammatory responses, oxidative stress, vascular dysfunction, endoplasmic reticulum stress, and the induction of both necrotic and apoptotic pathways (36). Despite the widespread clinical use of cisplatin and the emergence of various nephroprotection strategies, no universally accepted guidelines exist. Current practices largely emphasize adequate hydration and the avoidance of concurrent nephrotoxic agents (37). In preclinical models, researchers often employ time-course studies to dissect the temporal progression of kidney injury and to evaluate the effectiveness of novel therapeutic agents (38). These results highlight the complex pathophysiology of cisplatin-induced acute kidney injury and the lack of standardized preventative guidelines in clinical settings.

Kidney injury molecule-1 has emerged as a promising biomarker for detecting acute kidney injury and tubulointerstitial injury, including cases associated with anti-neutrophil cytoplasmic antibody (ANCA)-associated vasculitis and glomerulonephritis (39, 40). In humans, KIM-1 expression is predominantly localized to proximal tubular epithelial cells (41). A comprehensive meta-analysis encompassing 14 studies and over 3,300 adult patients demonstrated that urinary KIM-1 possesses strong predictive value for acute kidney injury, with notable sensitivity and specificity (42). Therefore, the results demonstrate the clinical relevance and diagnostic value of KIM-1 as a sensitive urinary biomarker for acute kidney injury. Emerging evidence supports the utility of KIM-1 in identifying cisplatin-induced renal damage across both in vitro and in vivo models (43). Kidney injury molecule-1 functions as a phosphatidylserine receptor, facilitating the phagocytosis of apoptotic bodies and necrotic debris by otherwise non-phagocytic epithelial cells (14, 44, 45). This unique capacity reprograms proximal tubular cells into semiprofessional phagocytes, a hallmark of renal stress response (14, 45). Cisplatin accumulation in these tubules promotes the formation of platinum complexes, which activate AMP-activated protein kinase (AMPK), subsequently reducing autophagy, enhancing DNA damage (46), increasing renal vascular resistance, and triggering tubular necrosis, inflammation, and apoptosis (47, 48). The results emphasize KIM-1's mechanistic role in renal injury and its potential as a functional biomarker that reflects tubular cell reprogramming and damage following cisplatin exposure. Taken together, these mechanistic insights align with our multi-level findings and further support the biological rationale for KIM-1 as a functional and diagnostic biomarker reflecting proximal tubular reprogramming and early injury following cisplatin exposure.

Notably, the time-course study by Jana et al. (49) demonstrated that urinary and tissue KIM-1 levels begin to rise as early as day 3 following cisplatin administration — well before elevations in serum creatinine and blood urea nitrogen (BUN) — paralleling the onset of tubular injury on histopathology. These findings reinforce our multi-level results showing rapid, proximal tubule–specific induction of KIM-1 prior to functional decline. Additional recent work supports these conclusions: Single-cell and spatial transcriptomic analyses (50, 51) confirm KIM-1’s restricted expression to injured proximal tubules, while mechanistic study (52) highlight its role in epithelial phagocytic reprogramming and stress-response signaling during nephrotoxic injury. Clinical studies (53, 54) further demonstrate that urinary KIM-1 offers superior early diagnostic performance compared with traditional markers in diverse acute kidney injury settings, including drug-induced nephrotoxicity. Integrating these recent findings broadens the contemporary context of our work and underscores that KIM-1’s early, dose-responsive, and mechanistically grounded induction is highly consistent across molecular, experimental, and clinical domains, thereby enhancing the relevance and novelty of our study.

### 5.1. Limitations

Despite the strengths of this study, some limitations should be considered when interpreting our findings. First, while the diagnostic accuracy results obtained from the meta-analysis provide a more reliable and evidence-based estimate than any individual study alone, the analysis was constrained by the small number of eligible clinical studies. This substantially reduces the statistical power of the pooled estimates and prevents subgroup analyses — such as stratification by cisplatin dose, timing of KIM-1 measurement, patient characteristics, or assay platform — that could offer more clinically nuanced insights. Future research should therefore prioritize high-quality, prospective clinical studies with standardized KIM-1 measurement protocols to enable more comprehensive and robust evidence synthesis. Second, although HAVCR1/KIM-1 demonstrated robust early induction across transcriptomic and experimental platforms, its clinical diagnostic performance was moderate. The meta-analysis showed an area under the curve of 0.76, indicating acceptable — but not optimal — accuracy for early detection of cisplatin-induced acute kidney injury. This suggests that while KIM-1 provides meaningful early diagnostic information, its performance may be further enhanced when used alongside additional complementary biomarkers. Accordingly, future research should explore multimarker strategies integrating KIM-1 with indicators such as neutrophil gelatinase-associated lipocalin (NGAL), interleukin-18 (IL-18), or cystatin C, as well as algorithmic or time-series modeling approaches that may improve early acute kidney injury detection. Finally, the methodological heterogeneity and risk of bias across the clinical studies further constrain the validity of the pooled results. The quality assessment of diagnostic accuracy studies-2 (QUADAS-2) identified a high risk of bias in the “index test” domain due to the frequent use of non-prespecified diagnostic thresholds for KIM-1. Variability in assay platforms, urine normalization practices, and timing of biomarker measurement also introduces inconsistency. To enhance reproducibility and clinical applicability, future studies should adopt predefined thresholds, standardized reporting frameworks, and rigorous prospective designs with appropriate blinding and validation.

### 5.2. Conclusions

Kidney injury molecule-1 (HAVCR1) is a proximal tubule-specific, injury-responsive marker that is rapidly and robustly induced at both the gene and protein levels following cisplatin exposure, reflecting early tubular damage. The findings establish KIM-1 as a clinically valuable early biomarker for detecting cisplatin-induced acute kidney injury, offering a non-invasive means for timely diagnosis and intervention to prevent progression of renal injury. Future studies should aim to validate KIM-1 performance across diverse patient populations and nephrotoxic conditions, establish standardized thresholds for clinical use, and investigate its utility in real-time monitoring of kidney health during chemotherapy.

ijpr-25-1-164432-s001.pdf

## Data Availability

Publicly available datasets analyzed in this study (single-cell RNA sequencing and bulk RNA sequencing) can be accessed through the Human Protein Atlas, GEO (accession number GSE227970), and the Susztak Lab Kidney Biobank. The raw data generated from the experimental studies are available from the corresponding author upon reasonable request.
