# Supplementary material for: KIM-1 as an Early Diagnostic Biomarker of Cisplatin-Induced Acute Kidney Injury
Source: Iran J Pharm Res. 2026 Feb 8;25(1):e164432. doi: 10.5812/ijpr-164432 (PMC13181219; doi:10.5812/ijpr-164432)
Supplement: ijpr-25-1-164432-s001.pdf [file ijpr-25-1-164432-s001.pdf]

Appendix 1

1. MEDLINE / PubMed (searched up to May 20, 2025)
Search string:
("HAVCR1"[Text Word] OR "KIM-1"[Text Word] OR "Kidney Injury Molecule-1"[Text Word])
AND
("Acute Kidney Injury"[MeSH Terms]
OR "acute kidney injury"[Text Word]
OR "acute renal injury"[Text Word]
OR AKI[Text Word])
AND
("Cisplatin"[MeSH Terms] OR cisplatin[Text Word])
Filters applied: None
Language restrictions: None

---

2. Scopus
Search string:
TITLE-ABS-KEY ("KIM-1" OR "Kidney Injury Molecule-1" OR HAVCR1)
AND
TITLE-ABS-KEY ("acute kidney injury" OR "acute renal injury" OR AKI)
AND
TITLE-ABS-KEY (cisplatin OR "cisplatin nephrotoxicity")
Document types: All
Language restrictions: None

---

3. Web of Science (Core Collection)
Search string (Topic Search – TS field):
TS=("KIM-1" OR "Kidney Injury Molecule-1" OR HAVCR1)
AND
TS=("acute kidney injury" OR "acute renal injury" OR AKI)
AND
TS=("cisplatin" OR "cisplatin nephrotoxicity")
Indexes searched: SCI-EXPANDED, SSCI, ESCI
Language restrictions: None

---

4. Cochrane Library
Search string (All Text / Keywords):
(KIM-1 OR "Kidney Injury Molecule-1" OR HAVCR1)
AND
("acute kidney injury" OR "acute renal injury" OR AKI)
AND
(cisplatin OR "cisplatin nephrotoxicity")
Filters: Trials, Reviews, Registered Studies (searched all)
Language restrictions: None

5. CNKI (China National Knowledge Infrastructure)
Search terms (translated and applied in Chinese & English):

(“KIM-1” OR “肾损伤分子1”)

AND

(“**急性**肾损伤” OR “**急性**肾衰竭”)

AND

(“顺铂”)

Language restrictions: None
